# The Function of Two *Brassica napus* β-Ketoacyl-CoA Synthases on the Fatty Acid Composition

**DOI:** 10.3390/plants14020202

**Published:** 2025-01-13

**Authors:** Dongfang Zhao, Bingqian Zhou, Bo Hong, Jiajun Mao, Hu Chen, Junjie Wu, Li Liao, Chunyun Guan, Mei Guan

**Affiliations:** 1 College of Agriculture, Hunan Agricultural University, Changsha 410128, China; zhaodongfang@stu.hunau.edu.cn (D.Z.); zhoubq202212@stu.hunau.edu.cn (B.Z.); hongbo@stu.hunau.edu.cn (B.H.); maojiajun26@gmail.com (J.M.); chenhu@stu.hunau.edu.cn (H.C.); goodboy-wjj@stu.hunau.edu.cn (J.W.); liaoli0412@stu.hunau.edu.cn (L.L.); guancy2001@hunau.edu.cn (C.G.); 2 Hunan Branch of National Oilseed Crops Improvement Center, Changsha 410128, China; 3 Southern Regional Collaborative Innovation Center for Grain and Oil Crops in China, Changsha 410128, China

**Keywords:** *Brassica napus* L., β-ketoacyl-CoA synthase (KCS), fatty acid composition

## Abstract

Rapeseed (*Brassica napus* L.) is one of the four major oilseed crops in the world and is rich in fatty acids. Changes in the fatty acid composition affect the quality of rapeseed. Fatty acids play various roles in plants, but the functions of the genes involved in the fatty acid composition during plant development remain unclear. β-Ketoacyl-CoA synthase (KCS) is a key enzyme involved in the elongation of fatty acids. Various types of fatty acid products are used to build lipid molecules, such as oils, suberin, wax, and membrane lipids. In *B. napus*, *BnaKCSA8* and *BnaKCSC3* belong to the KCS family, but their specific functions remain unclear. This study cloned *BnaKCSA8* and *BnaKCSC3* from *Brassica napus* L. and analyzed their functions. The gene structures of *BnaKCSA8* and *BnaKCSC3* were similar and they were localized to the endoplasmic reticulum (ER). In yeast, overexpression of *BnaKCSA8* increased the ratios of palmitoleic acid and oleic acid, while *BnaKCSC3* decreased the ratios of oleic acid. In Arabidopsis, overexpression of *BnaKCSA8* and *BnaKCSC3* lead to an increase in the proportion of linoleic acid and a decrease in the erucic acid. In summary, *BnaKCSA8* and *BnaKCSC3* altered the composition ratios of fatty acids. These findings lay the foundation for an understanding of the role of KCS in the fatty acids in rapeseed, potentially improving its health and nutritional qualities.

## 1. Introduction

Rapeseed (*Brassica napus* L.) is one of the four major oilseed crops globally and the largest oilseed crop in China [1,2]. Rapeseed oil is rich in fatty acids, including mainly palmitic acid (PA,C16:0), stearic acid (SA,C18:0), oleic acid (OA,C18:1), linoleic acid (LA,C18:2), linolenic acid (C18:3), eicosenoic acid (C20:0), erucic acid (C22:1), and nervonic acid (NA,C24:1). The composition of these fatty acids is an important indicator in evaluating the quality of rapeseed oil [3].

The extension of fatty acids in the endoplasmic reticulum occurs through a series of four consecutive reactions—condensation, reduction, dehydration, and a second reduction—adding two carbon atoms with each cycle [4]. The elongase complex consists of β-ketoacyl-CoA synthase (KCS), trans-2,3-enoyl-CoA reductase (ECR), 3-hydroxyacyl-CoA dehydratase (HCD), and 3-ketoacyl-CoA reductase (KCR) [5,6]. The *KCS* gene encodes β-ketoacyl-CoA synthase, which is a key enzyme that controls carbon chain elongation in the process of fatty acid biosynthesis [7].

KCS has been identified in various species, including Arabidopsis [8], rice [7], soybean [9], maize [10], peanut [11], and passion fruit [12]. However, there are variations in the members of the KCS gene family among different species. The influence of KCS family members on the composition of fatty acids can vary across different species [13,14,15,16]. In Arabidopsis, *AtKCS18/AtFAE1* is responsible for catalyzing the synthesis of erucic acid (C22:0) [17]. *AtKCS2* and *AtKCS20* are associated with changes in the fatty acid components from C20 to C22 [18]. *AtKCS10/AtFDH* primarily participates in the composition of fatty acids in the epidermal cells of flowers and young leaves [8,19,20]. *AtKCS17* is involved in changes in the fatty acid components from C22 to C24, and it interacts with *KCR1*, *PAS2*, and *ECR* [21]. *KCS* has also been studied in rapeseed. *KCS18* is a key gene in *Brassicaceae* plants, where it alters the erucic acid content in seed fatty acids [22,23]. *BnKCS1* can alter the wax composition in rapeseed leaves, reducing water loss and improving its drought resistance [24].

As a rate-limiting enzyme that catalyzes fatty acid elongation, the isolation and functional analysis of the *KCS* gene will help to elucidate the mechanisms behind changes in the fatty acid composition within plants. Although research on members of the KCS family has been conducted in many plant species, there has been limited research on rapeseed. Results regarding the regulatory network of high oleic acid in rapeseed lipid metabolism has indicated that *KCS* is involved [25]. In order to reveal the functions of the *BnaKCSes* in the fatty acid composition, this study cloned the full-length cDNA sequences of *BnaKCSA8* and *BnaKCSC3* and identified their functions. The functions of these two genes were preliminarily validated in yeast and Arabidopsis, laying the foundation for further in-depth research on rapeseed. This knowledge will contribute to a better understanding of the specific roles of *KCS* genes in the fatty acid composition in rapeseed, as well as offering new strategies for the development of functional rapeseed breeding.

## 2. Results

### 2.1. Bioinformatics Analysis of BnaKCSes

**Figure 1:**
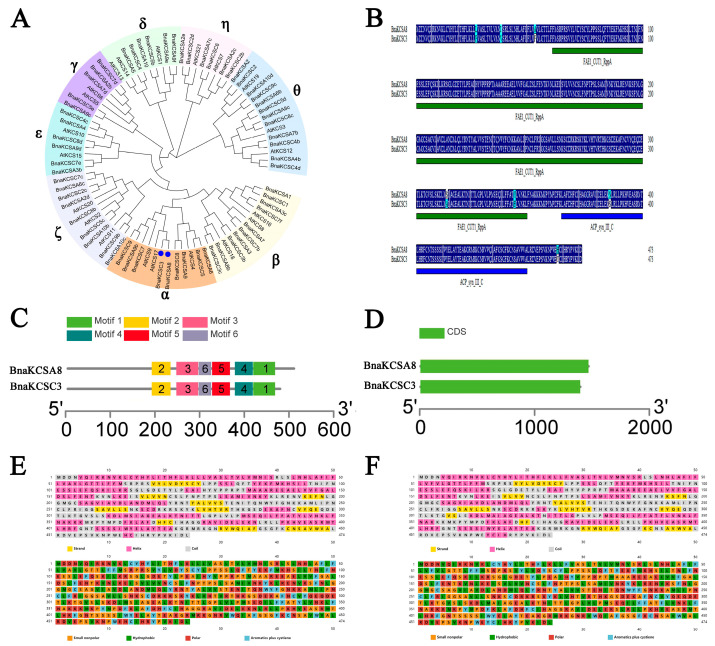
Bioinformatics analysis of *BnaKCSes*. (**A**) The phylogenetic tree constructed from 21 *AtKCS* family members and 63 *BnaKCS* members; dots: *BnaKCSA8* and *BnaKCSC3*. (**B**) Sequence alignment and conserved domain analysis of BnaKCSA8 and BnaKCSC3. (**C**,**D**) Gene structure and motif analysis of *BnaKCSA8* and *BnaKCSC3*. (**E**) Protein secondary structure prediction; yellow denotes strands, pink denotes helices, and gray denotes coils. (**F**) Properties of amino acids in BnaKCS protein; orange denotes small non-polar, green denotes hydrophobic, red denotes polar, and blue denotes aromatic compounds plus cysteine.

Using the *AtKCS* gene family sequences as a reference, we performed a BLAST search for *KCS* gene family members in the *B. napus* database. The results are shown in Figure 1A, and we identified 63 KCS gene family members in *B. napus*, which were divided into α-θ 8 subfamilies and distributed across 19 chromosomes. *BnaKCSA8* and *BnaKCSC3* were in the same group, indicating that they are homologous genes and may have similar functions.

The gene sequences of the *BnaKCSes* were determined by querying the reference genome website for rapeseed. The open reading frame (ORF) lengths of *BnaKCSA8* and *BnaKCSC3* were both 1425 bp, encoding 474 amino acids. BnaKCSA8 molecular weight was 53.78 kDa, theoretical pI was 9.35, the instability index (II) was computed to be 40.01, which classifies the protein as unstable. The grand average of hydropathicity (GRAVY) was −0.016, indicating that is hydrophilic protein. BnaKCSC3 molecular weight was 53.84 kDa, theoretical pI was 9.35, the instability index (II) was computed to be 38.83, which classifies the protein as stable. The GRAVY was 0.008, indicating that is hydrophobic protein. The conserved domains of the protein sequences of the KCSes were analyzed using the SMART website. The results indicated that both contain similarly positioned ACP_syn_III_C and FAE1_CUT1_RppA domains (Figure 1B). This indicates that BnaKCSA8 and BnaKCSC3 belong to the KCS family [26]. Through the analysis of the gene structures and motifs, it was found that the two rapeseed KCS members, *BnaKCSA8* and *BnaKCSC3*, are highly similar in terms of their gene structures. This similarity may reflect their close evolutionary relationship and suggests that they have similar roles and functions (Figure 1C,D).

The properties of the amino acids and secondary structure elements of the two genes are shown in Figure 1. Figure 1E represents BnaKCSA8, while Figure 1F represents BnaKCSC3. In the illustration of the amino acid properties, orange squares represent non-polar amino acids, green squares represent hydrophobic amino acids, red squares indicate hydrophilic amino acids, and blue squares denote aromatic compounds along with cysteine. In the protein secondary structure diagram, yellow squares represent strands, pink squares indicate helices, and gray squares denote coils (Figure 1E,F).

### 2.2. Gene Cloning and Subcellular Localization

**Figure 2:**
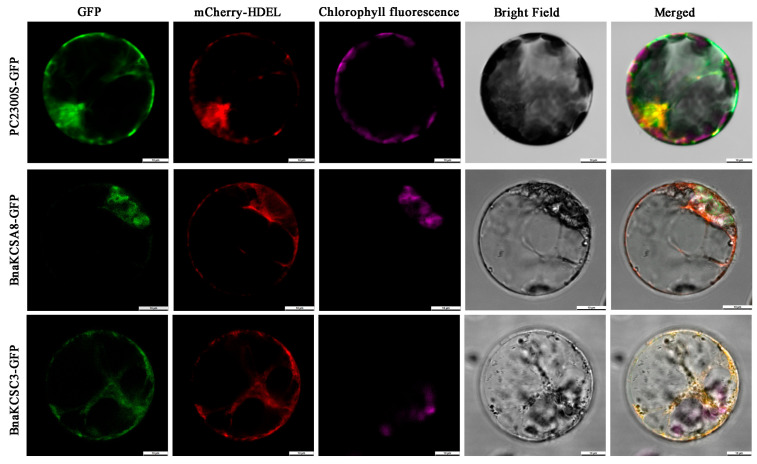
Subcellular localization of BnaKCSA8 and BnaKCSC3. Green is GFP (green fluorescent proteins) fluorescence, red is endoplasmic reticulum marker mCherry-HDEL, purple is chloroplast self-luminescence, PC2300S-GFP is empty vector control.

To study the subcellular localization of these two BnaKCS proteins, we cloned the two genes and fused them with the GFP (green fluorescent proteins) protein for expression. Co-expression was induced using PEG (polyethylene glycol) in Arabidopsis protoplasts along with endoplasmic reticulum (ER) markers. In the empty control, the GFP signals were distributed throughout the entire cell. The green fluorescent signals of BnaKCSA8 overlapped with the red ER marker, indicating that this gene was localized in the ER. BnaKCSC3 showed consistent localization signals with BnaKCSA8, with both proteins being localized in the ER (Figure 2). The ER is the site of fatty acid elongation, and these two genes are localized to the ER. Therefore, we speculate that they are involved in altering the fatty acid composition.

### 2.3. Expression Characteristics of BnaKCSes

**Figure 3:**
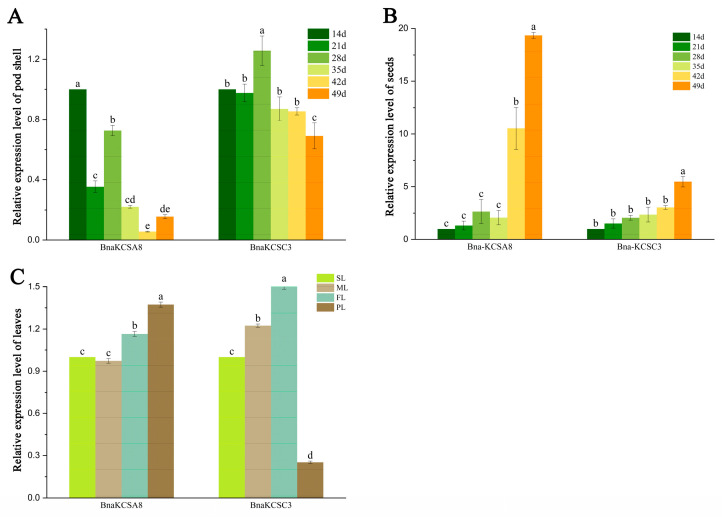
Expression characteristics of *BnaKCSes*. (**A**) Relative expression levels of *BnaKCSA8* and *BnaKCSC3* in pod shell; d: Seed development days. (**B**) Relative expression levels of *BnaKCSA8* and *BnaKCSC3* in seeds. (**C**) Relative expression levels of *BnaKCSA8* and *BnaKCSC3* in leaves; SL: seedling-stage leaves, BL: bolting-stage leaves, FL: flowering-stage leaves, PL: pod-stage leaves. Data are presented as the mean ± SE from three biological replicates. Different lowercase letters indicate a significant difference, as determined by Tukey’s test (*p* < 0.05).

To investigate the expression of the *BnaKCSA8* and *BnaKCSC3* genes in the leaves and pods during rapeseed development, we collected leaves and pods at different growth stages to analyze the gene expression levels. The expression of *BnaKCSes* in the podshell and seeds showed different trends, in podshell the expression levels of *BnaKCSes* showed a decreasing trend (Figure 3A). However, in seeds the expression of *BnaKCSA8* and *BnaKCSC3* gradually increased as they developed, reaching their highest levels in the seeds at 49 d, showing a marked difference compared to the levels at 14 d (Figure 3B). The expression levels of *BnaKCSA8* gradually increased in the leaves as they developed, reaching their highest levels in the pod stage; however, in the same time period, *BnaKCSC3* had the lowest expression (Figure 3C).

### 2.4. Function of BnaKCSes in Yeast

**Figure 4:**
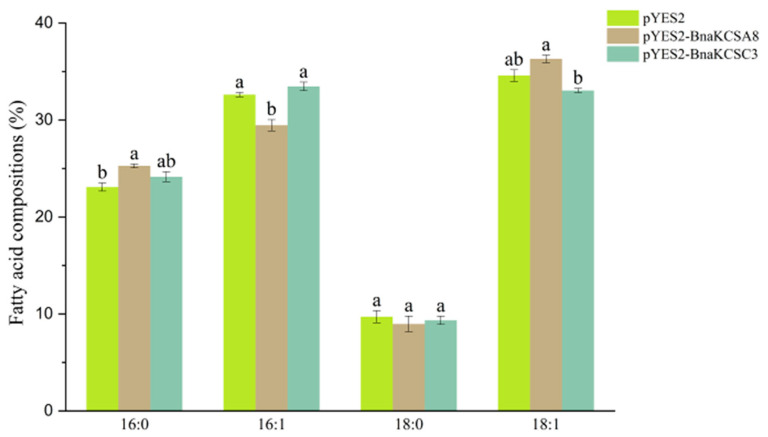
Effects of expressions of *BnaKCSA8* and *BnaKCSC8* genes on fatty acid composition in yeast. pYES2 represents the empty vector; pYES2-*BnaKCSA8* and pYES2-*BnaKCSC3* are the transgenic yeasts. Data are presented as the mean ± SE from three biological replicates. Different lowercase letters indicate a significant difference, as determined by Tukey’s test (*p* < 0.05).

To analyze the function of *BnaKCSA8* and *BnaKCSC3*, we first investigated whether they could alter the fatty acid composition in yeast cells. Compared to the empty vector pYES2, the pYES2-A8 significantly increased the level of PA (C16:0), decreased the level of C16:1, and reached a statistically significant level of difference, and the OA (C18:1) also increased, but not reach the level of significant difference. pYES2-C3 had a slight change in fatty acid composition, but the data did not reach the level of significant difference. This indicates that *BnaKCSA8* could change the composition of fatty acids, and *BnaKCSC3* has little effect on the composition of fatty acids (Figure 4).

### 2.5. Function of BnaKCSes in Arabidopsis

**Figure 5:**
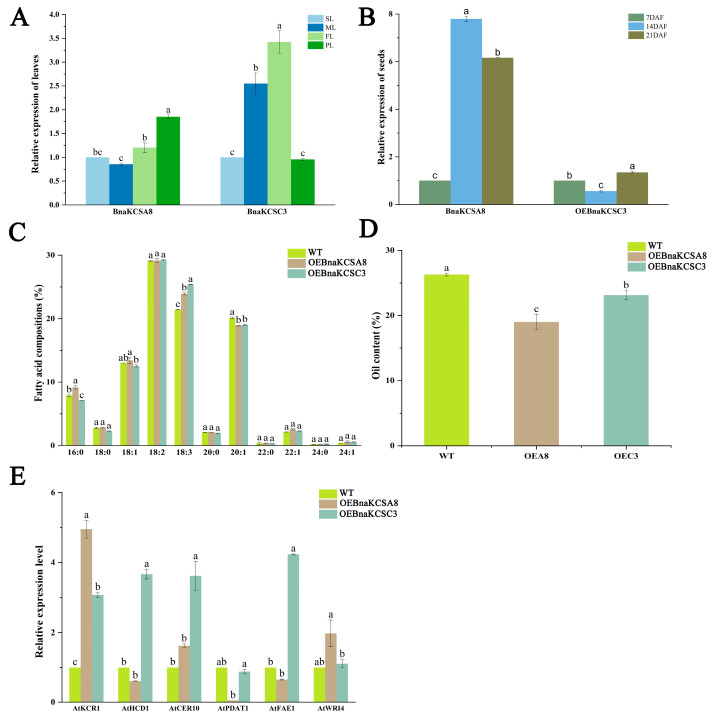
Analysis of gene expression and fatty acid composition. (**A**) Analysis of the relative expression of *BnaKCSes* in leaves. SL: seedling-stage leaves, BL: bolting-stage leaves, FL: flowering-stage leaves, PL: pod-stage leaves. (**B**) Analysis of the relative expression of *BnaKCSes* in seeds. DAF: days after flowering. (**C**) Fatty acid composition analysis of seeds; WT: wild-type, OE: overexpressing. (**D**) Oil content of seeds. (**E**) Expression analysis of genes related to lipids. Data are presented as the mean ± SE from three biological replicates. Different lowercase letters indicate a significant difference, as determined by Tukey’s test (*p* < 0.05).

To analyze the function of *BnaKCSA8* and *BnaKCSC3* in the fatty acid composition, we constructed two *BnaKCS* genes into a plant expression vector and performed genetic transformation in Arabidopsis. We selected leaves and seeds from different developmental stages for the analysis of the gene expression levels. The gene expression levels changed gradually over time. In the leaves, overexpressing the *BnaKCSA8* (OEA8) gradually increased over the growth period, reaching its peak in the leaves during the pod stage. However, in overexpressing the *BnaKCSC3* (OEC3), the expression level first increased and then decreased (Figure 5A). In the seeds, these strains exhibited the opposite trend compared to that in the leaves (Figure 5B). These results suggest that there is a spatiotemporal difference in the gene expression levels between the leaves and seeds.

To further determine the roles of *BnaKCSA8* and *BnaKCSC3* in the fatty acid composition, we also extracted seed fatty acids from transgenic Arabidopsis for GC-MS analysis (Figure 5C). Compared to the wild-type (WT) control, the fatty acid composition in the seeds of the transgenic lines showed varying degrees of change. In OEA8, the levels of PA (C16:0) increased by 16.3% and linolenic acid (C18:3) by 11.6%, the EA (C22:1) decreased by 5.5%. In OEC3, the levels of linolenic acid (C18:3) increased by 18.5%, in contrast, the levels of PA (C16:0) and EA (C22:1) decreased by 9.6% and 5.9%. This suggests that *BnaKCSA8* and *BnaKCSC3* altered the fatty acid composition. In addition, we measured the oil content of the seeds from each transgenic Arabidopsis line. As shown in Figure 5D, the oil content in all lines exhibited a decreasing trend compared to the wild type. This indicates that *BnaKCSA8* and *BnaKCSC3* might also have an impact on the oil content.

Moreover, we considered whether the transgenic modification would affect other lipid-related genes. We analyzed the expression levels of six lipid-related genes in 21 DAF seeds from both the wild-type and transgenic plants. The qRT-PCR analysis showed that *AtKCR1*, *AtCER10* (eceriferum), and *AtWRI4* (wrinkled) were upregulated in all transgenic lines (Figure 5E). In contrast, the expression levels of *AtHCD1* (hydroxyacyl-CoA dehydratase), *AtPDAT1* (phospholipid diacylglycerol acyltransferase), and *AtFAE1* (fatty acid elongation) were downregulated in OEA8. The results suggest that changes in the fatty acid composition may be regulated by multiple genes.

## 3. Discussion

With the promotion of functional plant oils, individuals are increasingly focusing on the health benefits and nutritional value of fatty acids [27,28]. This awareness could lead to more informed dietary choices that prioritize heart health and overall well-being [29,30,31,32,33]. The fatty acid composition of rapeseed has become a focal point for researchers in rapeseed breeding both domestically and internationally [34,35,36]. The fatty acid composition of seeds is regulated by a few key genes, but oil content involves multiple metabolic pathways [36]. This complexity highlights the challenges and opportunities in improving oilseed crops through genetic and metabolic engineering. Identifying the functions of genes involved in the fatty acid composition could provide an important theoretical basis for the breeding of functional seed oils. This knowledge could help to enhance the nutritional quality and health benefits of oilseeds, ultimately contributing to the development of improved varieties with desirable oil profiles.

*KCS* is a key gene that has been proven to participate in fatty acid elongation, and its products can alter the composition of fatty acids. The fatty acid composition of rapeseed is closely related to the quality of the seed oil, so it is essential to isolate and identify the functions of *BnaKCSes*. Therefore, two *BnaKCS* family members, *BnaKCSA8* and *BnaKCSC3*, were cloned and identified in *Brassica napus*. Through genetic evolution and bioinformatics analyses, it has been found that these two members have strong similarity and belong to the α-group of the KCS family, indicating a close phylogenetic relationship. These two members encode 474 aa. An analysis of the protein domains of the BnaKCSes through the SMART database revealed that they both contained similarly located ACP_syn_III_C and FAE1_CUT1_RppA domains (Figure 1B). This is consistent with the findings of Sagar regarding the conserved domains of KCS family proteins, indicating that the two genes identified in this study belong to the KCS gene family [26]. The gene structure and motif analysis results show that both genes contain the same motifs, and the number of introns and exons is consistent (Figure 1C–D). Given the striking similarity in the structures of these two genes, it is inferred that they may play similar roles in fatty acid biosynthesis. We also conducted subcellular localization studies on *BnaKCSA8* and *BnaKCSC3* using Arabidopsis protoplasts. The results show that both genes are localized in the endoplasmic reticulum (Figure 2), which is consistent with the subcellular localization results for *KCS* [21].

To investigate the roles of these two *BnaKCSes* in the fatty acid composition, we first conducted preliminary validation in yeast cells to determine whether the *BnaKCSes* could alter the composition of fatty acids. Compared to the control, the content of OA (C18:1) and PA (C16:0) were significantly increased in the pYES2-A8 transgenic yeast cells. In the pYES2-C3 transgenic yeast cells, the content of OA (C18:1) decreased (Figure 4), indicating that both genes are capable of altering the fatty acid composition. This is consistent with the results observed for peanut *AhKCS*, which also changed the composition of fatty acids when expressed in yeast [11]. In *Camelina sativa*, the heterologous expression of the *Lunaria annua KCS* also altered the fatty acid composition [37]. Additionally, we overexpressed the *BnaKCSes* in Arabidopsis to further validate their function. In OEA8, the levels of PA (C16:0) increased by 16.3% and linolenic acid (C18:3) by 11.6%, EA (C22:1) decreased by 5.5% (Figure 5C). This indicates that the introduction of *BnaKCSA8* altered the composition of fatty acids. This is in line with results showing that *KCS* can alter the fatty acid composition in Arabidopsis seeds [17,38]. In OEC3, the levels of linolenic acid (C18:3) increased by 18.5%, in contrast, the levels of PA (C16:0) and EA (C22:1) decreased by 9.6% and 5.9% (Figure 5C). This is consistent with results regarding the overexpression of *CsKCS6*, which led to changes in the fatty acid composition [39]. The fatty acid components altered by *BnaKCSes* in yeast and Arabidopsis were different, which may be due to the difference in the substrate required by these two genes, or some other reasons, which need further study. In this study, these two genes can change the fatty acid composition. Oil content analysis showed that the oil content of transgenic plants was lower than that of wild type (Figure 5D), which was similar to the results of BnFAE1 [23]. Although overexpression of a gene involved in fatty acid biosynthesis is expected to increase oil content. However, because the oil content is affected by many factors, the changes of fatty acid components are not necessarily positively correlated with the oil content.

Research by Kim et al. has shown that *KCS* interacts with *KCR1*, *PAS2*, and *ECR* to regulate fatty acids [21]. To determine whether other genes respond to fatty acids, we also examined the expression levels of additional genes associated with fatty acids in transgenic plants. Compared to the WT, *AtKCR1*, *AtCER10*, and *AtWRI4* were upregulated in the OEA8 line (Figure 5E). This result is consistent with the findings that the overexpression of *BnWRI1* increases the fatty acid content and that the upregulation of *AtWRI* transcription factors promotes oil accumulation [40,41].

## 4. Materials and Methods

### 4.1. Plant Materials and Growth Conditions

In this study, the rapeseed considered was *Brassica napus* L. “Gaoyousuan No.1”. It was developed by academician Guan Chunyun from Hunan Agricultural University.

Plants were grown under a photoperiod consisting of 16 h light/8 h darkness. Soil-grown *A. thaliana* plants were cultured in pots containing a well-mixed organic substrate and vermiculite (3:1; *v*/*v*) and irrigated with water every three days and with half-strength Hoagland nutrient solution once a week. The relative humidity of the culture chamber was 60%, and the temperature was 21 °C.

### 4.2. Bioinformatics Analysis of BnaKCSes

The Arabidopsis family sequence was downloaded from the TAIR website (https://www.arabidopsis.org/, accessed on 8 January 2023), and the *B. napus* reference genome data were downloaded from BRAD (http://brassicadb.cn/#/, accessed on 8 January 2023). Phylogenetic evolutionary analysis was performed with MEGA_6. For the protein conserved domain analysis, we utilized the SMART online tool (https://smart.embl.de/, accessed on 8 January 2023). The conserved domain images were generated using the DNAMAN software, and the protein motifs were analyzed using the online MEME tool (https://meme-suite.org/meme/, accessed on 8 January 2023). The physical and chemical property analysis and primary structure analysis were obtained from Expasy (https://web.expasy.org/protparam/, accessed on 8 January 2023). The amino acid sequences of the proteins were analyzed regarding their secondary structures through PSIPRED (http://bioinf.cs.ucl.ac.uk/, accessed on 8 January 2023).

### 4.3. BnaKCS Genes Cloning

The primer sequence referred to the reference sequence from the *B. napus* genome database. The total RNA was extracted using the FastPure Universal Plant Total RNA Isolation Kit, according to the manufacturer’s instructions (Vazyme, Nanjing, China). RNA reverse transcription was performed using the HiScript II 1st Strand cDNA Synthesis Kit (Vazyme, China).

*BnaKCSA8* (BnaA08G0134800ZS) and *BnaKCSC3* (BnaC03G0746000ZS) were cloned from the cDNA template using the Phanta Flash Master Mix (Vazyme, China). The cloned product sequence was transformed into *E. coli*, and the full-length sequence was determined after sequencing and comparison. The cloned sequences were transformed into *E. coli*, and Sanger sequencing was performed to determine the sequence. The cloning primers are listed in Supplementary Table S1.

### 4.4. Subcellular Localization

To determine the subcellular localization of the BnaKCSA8 and BnaKCSC3 proteins, we transiently expressed BnaKCSA8 and BnaKCSC3 in fusion with green fluorescent protein (GFP) as a fluorescent marker under the control of the CaMV 35S promoter in *A. thaliana* protoplasts. The PCR product was transformed into the pCAMIBA2300-GFP vector carrying the CaMV 35S promoter for N-terminal GFP fusion using the ClonExpress-II One Step Cloning Kit (Vazyme, China), according to the manufacturer’s protocol. The well-established fluorescent protein marker mCherry-HDEL was used for the ER [42]. The transient expression of BnaKCSes-GFP and the marker protein in *A. thaliana* mesophyll protoplasts was performed by following a published method [43]. The transformed *A. thaliana* protoplasts were observed with a confocal laser scanning microscope. The empty vector pCAMIBA2300-GFP was analyzed as a control.

### 4.5. Yeast Cell Transformation

The *BnaKCS* gene was integrated into the yeast expression vector PYES2/NT and genetically transformed into the Saccharomyces cerevisiae strain InvSc1 (Invitrogen). A yeast transformed with the empty vector PYES2/NT served as a negative control. The yeast induction method was derived from Xue [44]. Yeast cells were grown in SC-ura medium supplemented with 2% galactose. The culture was incubated overnight at 28 °C until an OD600 of 1.5 was reached.

### 4.6. Overexpression of BnaKCSes in A. thaliana

The full-length coding sequence of *BnaKCS* was cloned into the pCAMIBA1301 vector under the control of the CaMV 35S promoter. The vector pCAMIBA1301-BnaKCS was introduced into EHA105, and Col-0 wild-type plants were transformed into Arabidopsis thaliana by flower impregnation method at full flowering stage [45]. Transgenic plants were screened based on their resistance to hygromycin. T3-generation transgenic plants were used for the subsequent validation experiments.

### 4.7. Lipid Extraction

The preparation and detection of fatty acid methyl esters from yeast cells was performed in accordance with Katavic [46]. Overnight-cultured cells were collected via centrifugation and washed with ultra-pure water. The yeast cells were saponified in a potassium hydroxide–methanol solution (10% (*w*/*v*) KOH, 5% (*v*/*v*) in methanol) at 80 °C for 2 h. After saponification, the samples were placed on ice and washed with n-hexane to remove unsaponified material. The remaining aqueous phase was acidified with 6 M HCl. The free fatty acids (FFAs) were extracted into n-hexane, and the solvent was evaporated under a nitrogen flow. The FFAs were then methylated for 2 h at 80 °C using 3 M sulfuric acid in methanol. The fatty acid methyl esters (FAMEs) were extracted in hexane, and the solvent was removed under a nitrogen flow. The residuals were dissolved in n-hexane. The induced expression and fatty acid analyses of the transgenic yeast were performed with three biological replicates.

The methods for the analysis and detection of fatty acids (FAs) were conducted following a previous study [47], using the Agilent 7890B gas chromatograph (GC). In particular, 0.2 g of mature seeds (dry weight, DW) was ground and added to a 5 mL glass tube containing 2 mL of a petroleum ether and ether solution (1:1). This was mixed and shaken for 10 min; then, it was left to stand for 40 min. After this, 2 mL of KOH methanol solution (0.4 mol/L) was added and the sample was mixed. The methanol transesterification reaction lasted 30 min; this was followed by the addition of 2 mL of distilled water along the wall of the tube. Then, the mixture was shaken well and left it stand, and then the mixture was removed. Finally, 1 mL of the supernatant was collected and transferred into a GC vial for analysis. The corresponding peaks of each fatty acid (FA) were identified based on their characteristic retention times. The gas chromatographic analysis method is referred to Li [48]. The results were determined using the peak area normalization method, and the mass percentage (m%) of each component relative to the total FA was calculated. The oil content was determined using a Soxhlet extraction apparatus, employing petroleum ether extraction (GB/T 2906-1982).

### 4.8. RNA Extraction and Quantitative Real-Time Polymerase Chain Reaction

In both rapeseed and Arabidopsis, leaves were sampled during the seedling-stage, bolting-stage, flowering-stage, and pod-stage, with collections made every 7 days throughout the seed growth period. Fresh leaves and seeds were frozen in liquid nitrogen, and the total RNA was extracted using the FastPure Universal Plant Total RNA Isolation Kit (Vazyme, China). The RNA was then reverse-transcribed into cDNA using the HiScript II 1st Strand cDNA Synthesis Kit (Vazyme, China).

The spatiotemporal expression of *B. napus KCS* genes was examined via a quantitative real-time polymerase chain reaction (qRT-PCR) using the Taq Pro Universal SYBR qPCR Master Mix (Vazyme, China). The primer sequences are listed in Table S2. *AtActinβ* and *BnaActin* were used as internal references. The relative expression levels of the *KCSes* were calculated using the 2−ΔΔCT method [49]. All expression analyses consisted of three biological and three technical replicates.

### 4.9. Data Presentation and Statistical Analysis

Data analysis and bar chart plotting were performed using the Origin (2021) software. In this study, each experimental design included at least three biological replicates (*n* ≥ 3), with the values presented as the mean ± standard deviation. Tukey’s test was used, and a *p*-value of ≤0.05 was considered statistically significant.

## 5. Conclusions

In summary, in the present study, we cloned two *BnaKCS* members, both of which can alter the composition of fatty acids in yeast and Arabidopsis. In the yeast, both *BnaKCSes* changed the fatty acid composition and ratios. In Arabidopsis, a similar change in the fatty acid composition was observed in the seeds. While the heterologous expression results obtained in yeast and Arabidopsis indicate that *BnaKCSA8* and *BnaKCSC3* can alter the fatty acid composition, further research is needed to determine whether they have the same function in rapeseed. These findings provide theoretical support for an understanding of the molecular mechanisms associated with the fatty acid composition and offer candidate genes for functional rapeseed breeding.

## Data Availability

All the data included in this study are available upon request by contacting the corresponding author.

## Supplementary Materials

The following supporting information can be downloaded at: https://www.mdpi.com/article/10.3390/plants14020202/s1, Table S1: Gene Primers; Table S2: Primers for gene expression.
