# Supplementary material for: The Function of Two Brassica napus β-Ketoacyl-CoA Synthases on the Fatty Acid Composition
Source: Plants (Basel). 2025 Jan 13;14(2):202. doi: 10.3390/plants14020202 (PMC11769367; doi:10.3390/plants14020202)
Supplement: Supplementary file 1 [file plants-14-00202-s001.zip › plants-3369663-supplementary.pdf]

Supplementary Table S1. Gene primers

Primer Name	Sequence
A08-13480-F	ATGGACGACAACGTACAG
A08-13480-R	TCAGAGATCTATCTTAACAGG
C03-74600-F	ATGGACGACAACGTACAG
C03-74600-R	TCAGAGATCAATCTTAACG
C3-GFP-F	GCTTTCGCGAGCTCGGTACCATGGACGACAACGTACAG A
C3-GFP-R	CCCTTGCTCACCATGGATCCGAGATCAATCTTAACGGGA
A8-GFP-F	GCTTTCGCGAGCTCGGTACCATGGACGACAACGTACAG A
A8-GFP-R	CCCTTGCTCACCATGGATCCGAGATCTATCTTAACAGGA
INV-BnaA8-F	CGGGGTACC ATGGACGACAACGTACAG
INV-BnaA8-R	TGCTCTAGA GAGATCTATCTTAACAGG
INV-BnaC3-F	CGGGGTACC ATGGACGACAACGTACAG
INV-BnaC3-R	TGCTCTAGA GAGATCAATCTTAACG
OE-BnaA8-F	CATGCCATGG ATGGACGACAACGTACAG
OE-BnaA8-R	CGGACTAGT TCAGAGATCTATCTTAACAGG
OE-BnaC3-F	CATGCCATGG ATGGACGACAACGTACAG
OE-BnaC3-R	CGGACTAGT GAGATCAATCTTAACG

Supplementary Table S2. Primers for gene expression

Primer Name	Sequence
β-ACTIN-F	AGTGGTCGTACAACCGGTATTGT
β-ACTIN-R	GAGGATAGCATGTGGAAGTGAGAA
Bn-Actin-F	GGTTGGGATGGACCAGAAGG
Bn-Actin-R	TCAGGAGCAATACGGAGC
q-BnaC3-F	ACCACTTTGGGTCCTCT
q-BnaC3-R	TGTTCCCTTTCCTCATT
q-AtFAE1-F	TTCAAGAGCGTTCAGGTC
q-AtFAE1-R	AGCGGATAGCGAAGGAGT
q-AtWRI4-F	TCCCTTTGCCGAGTTATG
q-AtWRI4-R	TCTTCTTGCGTGGCGTAT
q-AtKCR1-F	TGCTGCTGCTCTTATTCC
q-AtKCR1-R	GTAACCCTCTGGTGATGC
q-AtHCD1-F	CTCCAATGGCTTTGACCT
q-AtHCD1-R	ACCAACGCCGAGAAGTAG
q-AtCER10-R	GTTCAGCCACGCAACCTC
q-AtCER10-R	CCCGAAACCAATCTTCATCT
q-AtPDAT1-F	CTGGTTAGACGAATGGGTAG
q-AtPDAT1-R	TGACTTTGCCTTTGCTGA
